# Matrix factorization with neural network for predicting circRNA-RBP interactions

**DOI:** 10.1186/s12859-020-3514-x

**Published:** 2020-06-05

**Authors:** Zhengfeng Wang, Xiujuan Lei

**Affiliations:** 1 School of Computer Science, Shaanxi Normal University, Xi’an, 710119 China; 2 College of Information Science and Engineering, Guilin University of Technology, Guilin, 541004 China

**Keywords:** circRNA, RNA binding protein, Matrix factorization, Neural networks, Positive unlabeled learning

## Abstract

**Background:**

Circular RNA (circRNA) has been extensively identified in cells and tissues, and plays crucial roles in human diseases and biological processes. circRNA could act as dynamic scaffolding molecules that modulate protein-protein interactions. The interactions between circRNA and RNA Binding Proteins (RBPs) are also deemed to an essential element underlying the functions of circRNA. Considering cost-heavy and labor-intensive aspects of these biological experimental technologies, instead, the high-throughput experimental data has enabled the large-scale prediction and analysis of circRNA-RBP interactions.

**Results:**

A computational framework is constructed by employing Positive Unlabeled learning (P-U learning) to predict unknown circRNA-RBP interaction pairs with kernel model MFNN (Matrix Factorization with Neural Networks). The neural network is employed to extract the latent factors of circRNA and RBP in the interaction matrix, the P-U learning strategy is applied to alleviate the imbalanced characteristics of data samples and predict unknown interaction pairs. For this purpose, the known circRNA-RBP interaction data samples are collected from the circRNAs in cancer cell lines database (CircRic), and the circRNA-RBP interaction matrix is constructed as the input of the model. The experimental results show that kernel MFNN outperforms the other deep kernel models. Interestingly, it is found that the deeper of hidden layers in neural network framework does not mean the better in our model. Finally, the unlabeled interactions are scored using P-U learning with MFNN kernel, and the predicted interaction pairs are matched to the known interactions database. The results indicate that our method is an effective model to analyze the circRNA-RBP interactions.

**Conclusion:**

For a poorly studied circRNA-RBP interactions, we design a prediction framework only based on interaction matrix by employing matrix factorization and neural network. We demonstrate that MFNN achieves higher prediction accuracy, and it is an effective method.

## Background

Circular RNA (circRNA) is a novel type of non-coding RNAs which has covalent and closed loop structures. Compared to linear RNA, circRNA is more stable in cells [1]. It is generated through a non-sequential back-splicing process, in which a downstream 5′ splice donor back-splices to an upstream splice acceptor, and this process is regulated by both *cis* elements and *trans* protein factors [2]. For instance, some RNA Binding Proteins (RBPs) can enhance the formation of circRNA such as QKI and MBL [3, 4]. Inversely, some RBPs (e.g., PTBP1) can reduce circRNA formation [5, 6]. In recent years, with the development of high-throughput experimental for non-polyadenylated RNA transcripts, abundance and diversity of circRNA have been successfully discovered in various species [7], however, the biological functions of circRNA remain largely unknown. Emerging evidence has shown that circRNA plays an important role in human diseases, especially in cancers [8, 9]. Recent studies have reported that circRNA could promote cell proliferation [10–12] and serve as biomarkers in cancer [13, 14]. Several databases have been constructed to benefit the studies on links between circRNAs and human diseases, such as the circRNAs in cancer cell lines database (CircRic) [15] and the cancer-specific circRNA database (CSCD) [16]. CircR2Disease [17] curates a database for associations which are experimentally supported between circRNAs and diseases, and provides a platform for investigating mechanism of the disease-related circRNAs.

Increased evidence indicates that many circRNAs are interacting with RBPs [18], for instance, ciR-7/CDR1as is widely associated with Argonaute (AGO) proteins [19] and the circRNA MBL/MBNL1 contains conserved muscleblind (MBL) proteins binding sites [3]. Furthermore, the circRNA circPABPN1 could bind to HuR to prevent HuR binding to PABPN1 mRNA and lower PABPN1 translation [20]. In addition, although emerging evidence indicates that several circRNAs are translatable [21–23], the majority of circRNAs are not translated as linear mRNAs are. Therefore, RBPs bound to circRNAs are not replaced by ribosomes [24, 25]. Some databases have been developed for exploring the links between circRNAs and RBPs, such as CSCD provides miRNA target sites, RBPs binding sites and potential open reading frames (ORFs) in cancer-specific circRNAs. CircRic systematically characterizes circRNAs expression profile in 935 cancer cell lines across 22 cancer lineages, and analyzes the associations between circRNAs with mRNA, protein and mutation. starBase [26] systematically identifies the RNA-RNA and protein-RNA interaction networks from 108 CLIP-Seq data sets generated by 37 independent studies. Moreover, CircInteractome [27] provides bioinformatic analyses of binding sites on circRNAs and additionally analyzes miRNA and RBP sites on junction and junction-flanking sequences.

Thus, it is very meaningful to study the interaction between circRNAs and RBPs in cancer. To this day, these interactions are mainly analyzed by RNA immunoprecipitation (RIP) [28] or RNA pull-down assay [29]. The RNA is pulled-down by the probe for analyzing associated proteins in the RNA pull-down assay. In the RIP assay, a protein is immunoprecipitated for analyzing associated RNA. Although many significant discoveries have been made through these methods, it still faces some challenges such as cost-heavy, labor-intensive and time-consuming. Therefore, it is necessary to design a powerful computational method for predicting circRNA-RBP interactions, which further provides an important assistance for revealing the biological functions of circRNA. Computational prediction of circRNA-RBP interaction relationship could be divided into prediction of binding sites and interaction pairs. For example, CRIP [30] and CSCRSites [31] identify the binding sites on circRNA employing different deep learning methods, respectively. CircSLNN [32] identifies the specific location of RBP-binding sites on circRNAs by modeling the prediction of binding sites on RNAs as a sequence labeling problem. Several computational methods have been developed to predict lncRNA-protein interaction relationships [33–35]. To our knowledge, computational methods for predicting the circRNA-RBP interaction pairs have not been reported yet. In this study, we will focus on the problem of interaction pair’s prediction.

In the fields of link prediction, matrix factorization (MF) is the most popular and effective method which characterizes interaction pairs by vectors of latent factors [36]. Thereby this problem is modelled to the inner product of their latent vectors. More research effort has been devoted to extract latent vectors. Recently, neural network has been employed for obtaining the latent factors. Neural network-based Collaborative Filtering (NCF) leveraged a multi-layer perceptron to learn the interaction pairs function [37]. Xue et al. proposed a matrix factorization model with neural network architecture for top-N recommendation [38]. However, there is often a lack of reliable negative samples during training model. This problem is often referred as Positive-Unlabeled learning (P-U learning). Mordelet et al. designed a method which iteratively trains many classifiers model to discriminate the known positive examples from random subsamples of the unlabeled set, and averages their predictions [39].

Inspired by these research results, we designed a computational framework, matrix factorization based on neural network (MFNN) kernel model, to predict unknown circRNA-RBP interaction pairs with P-U learning. Here, neural network is employed to extract the latent factors of circRNA and RBP, then the P-U learning strategy is applied to predict unknown interaction pairs. In addition, there are still no unified public datasets on circRNA-RBP interaction, especially in human cancer. Therefore, we construct the circRNA-RBP interaction matrix using the data in CircRic database [15]. The experimental results show that MFNN kernel outperforms the other deep kernel model. Moreover, we score the unlabeled interactions pairs using P-U learning, and match the predicted interactions to the known interactions database, which indicate that our method is effective in analyzing the circRNA-RBP interactions.

## Results

In this section, in order to assess the validity of the prediction results, various validation methods are employed to evaluate the MFNN model. It is also compared with some existing representative matrix factorization based on deep learning. Finally, we scored the unlabeled interactions pairs, and matched the predicted interaction pairs to the known interaction databases, indicating that our method is effective in analyzing the circRNA-RBP interactions.

### Experimental and hyper-parameters settings

The MFNN is implemented in python 3.7 by using TensorFlow 1.14.0 library. To determine parameters of the designed model, during the experiments, P-U learning strategy is adopted to build the training set and the negative samples are sampled from unlabeled samples each time, which has the same number as the positive samples. In training phase, the batch size is set to 256, and learning rate is 0.0001. Finally, the Adam optimizer is employed to optimize the model. In addition, to further evaluate the prediction model, cross validation is applied to assess the performance of the prediction model [40]. In this study, 10 times 10-fold cross validation and 10 times 5-fold cross validation are employed to evaluate the prediction model. The training set is divided into two groups with randomly sampling (90% for training and 10% for validating). This process is repeated 10 times. Ten times 5-fold cross validation is similar. Inspired by the idea of P-U learning algorithm, the 10 times CV-5 and CV-10 are different from classical method in this study, in each time, the negative samples are selected from the unlabeled sample set randomly, and generate the new training set. The different validation settings are analyzed for the CRI model as follows:

CV-10: During the model training, the training set are divided into 10 folds, in each round, one-fold is regarded as validating data and the remaining data as training data. This process is repeated 10 times.

CV-5: Like the CV-10-fold, the training set are divided into five folds, in each round, one-fold is regarded as test data and the remaining data as training data. This process is repeated 10 times.

### Depth of layers in neural network

In the MFNN model, the low-dimensional latent factors of circRNA and RBP are extracted through neural network. Usually, the architecture of neural network has a significant impact on its performance, especially the depth of network is a prominent impact factor. In this section, we studied the different depths of network and the different combinations of latent factors in each layer, and selected the parameters with the best performance. Here, the area under the receiver operating characteristics curve (AUC) and the area under the precision-recall curve (AUCPR) are used as metric for model evaluation.

**Fig. 1:**
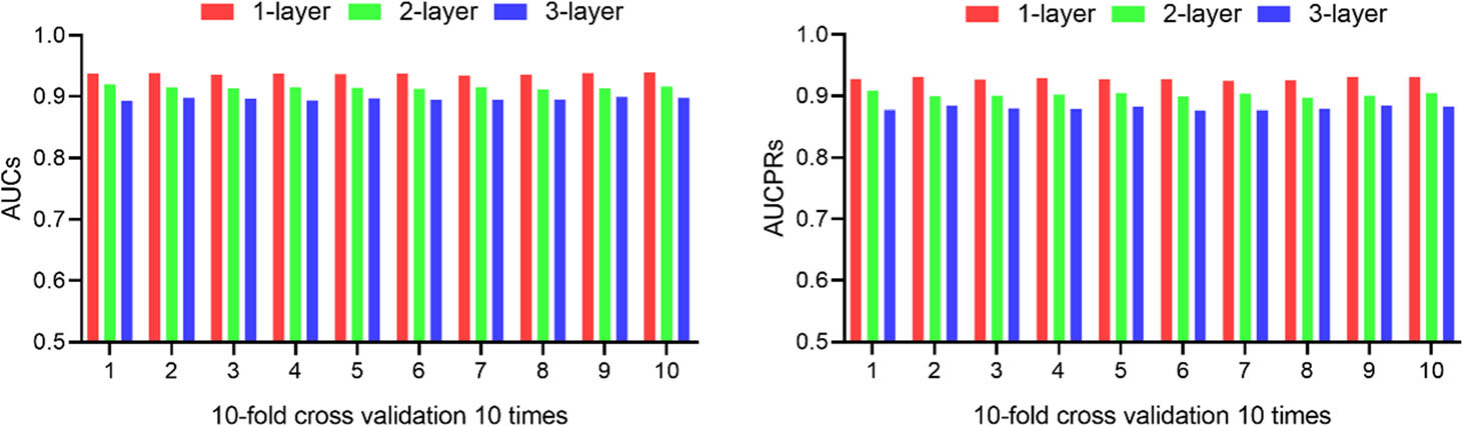
AUCs and AUCPRs of different network depths

First, different number of hidden layers are investigated, the AUCs and AUCPRs are compared with 10 times CV-10. The simulation results are shown in Fig. 1. Interestingly, the 1-layer achieves the best performance. The deeper of hidden layers does not mean the better, the 3-layers decreases the model performance. Finally, 1-layer network is adopted in MFNN.

**Fig. 2:**
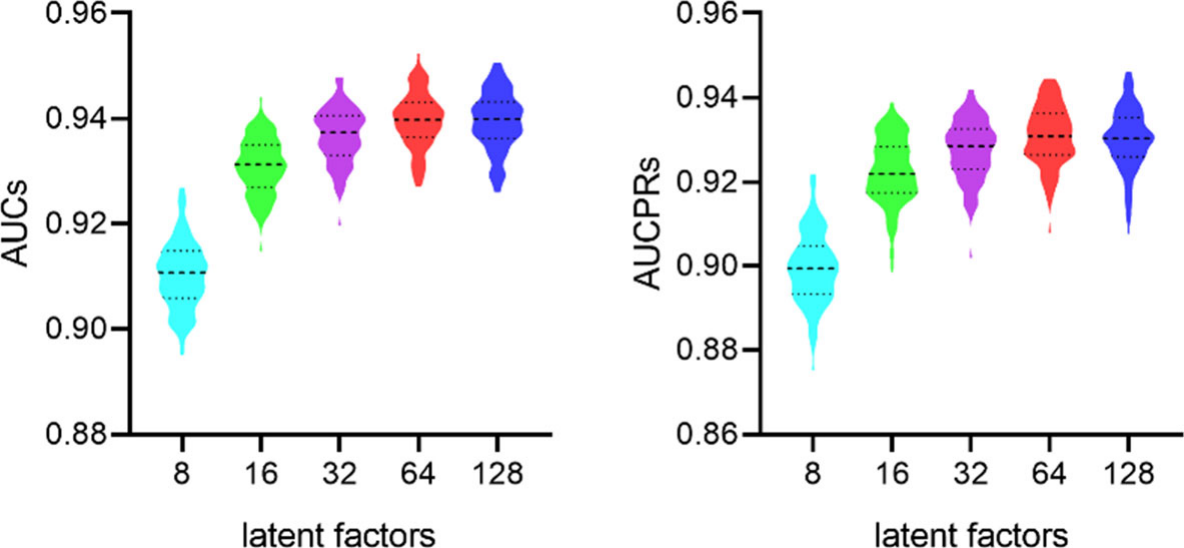
AUCs and AUCPRs of different latent factors

Moreover, the neuron numbers in each layer is possibly another sensitive parameter in neural networks. In MFNN, the neuron numbers are the latent factors of circRNA and RBP on the final layer, the performance with different numbers of neurons on the final layer are compared with 10 times CV-10, setting the numbers of neurons from 8 to 128. The average values of 10 times CV-10 in terms of AUC and AUCPR are shown in Fig. 2.

As shown in Fig. 2, the latent factors with 32, 64 and 128 achieve the better performance, and the AUC and AUCPR with latent factors 128 have no significant increase. Finally, the latent factors are set to 64.

### Performance evaluate

In this section, we introduce several evaluation metrics to comprehensively assess the performance of MFNN, such as sensitivity (*S*_*n*_), specificity (*S*_*p*_), precision (*P*_*r*_), accuracy (*Acc*) and Matthew’s correlation coefficient (*MCC*). They are defined as follows:

1 $$ {S}_n=\frac{TP}{T\mathrm{P}+ FN} $$

2 $$ {S}_p=\frac{TN}{TN+ FP} $$

3 $$ {P}_r=\frac{TP}{TP+ FP} $$

4 $$ Acc=\frac{TN+ TP}{TN+ FP+ TP+ FN} $$

5 $$ MCC=\frac{TP\times TN- FP\times FN}{\sqrt{\left( TP+ FP\right)\times \left( TN+ FN\right)\times \left( TP+ FN\right)\times \left( TN+ FP\right)}} $$

where *TP* and *TN* indicate the numbers of correctly predicted circRNA-RBP interaction pairs and non-interaction pairs, respectively. *FP* and *FN* are the numbers of incorrectly predicted circRNA-RBP interaction pairs and non-interaction pairs, respectively. In addition, various validation methods including CV-10 and CV-5 are employed to evaluate MFNN.

**Fig. 3:**
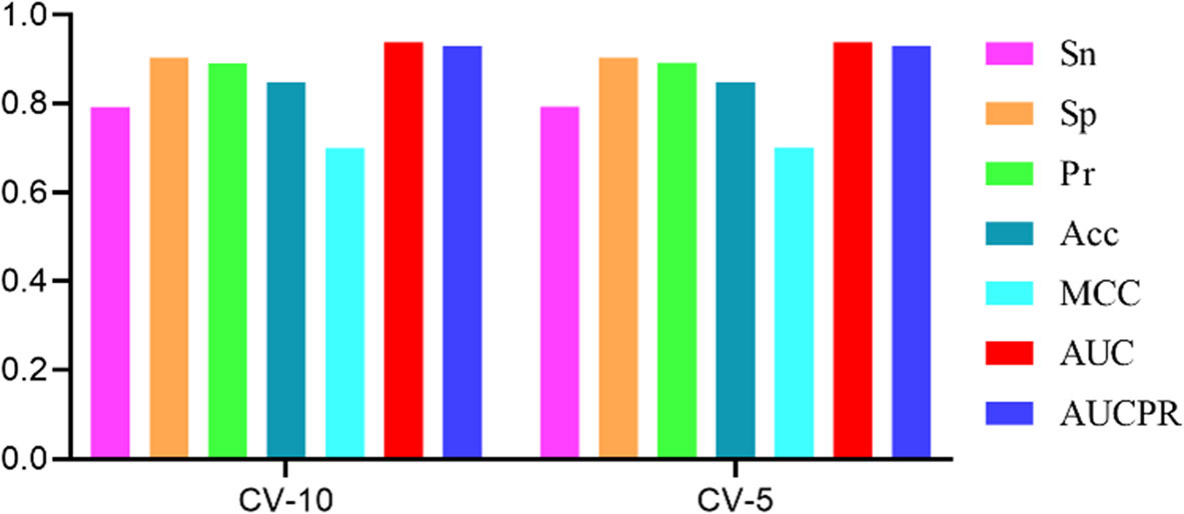
Comparison for MFNN with different validation methods

The performance of MFNN on each evaluation metric is shown in Fig. 3 with 10 times CV-10 and CV-5 validation methods, respectively. There is no significant difference in the evaluation metrics of the two validation methods. MFNN achieves the higher AUC and AUCPR values with two kinds of validation methods. The values of the other evaluation metrics usually depend on threshold in binary classification problem. Here, the threshold is set to 0.5 which means that the circRNA and RBP may interact when scores are more than 0.5, otherwise not.

### Performance comparison

He et al. present a neural architecture NCF (Neural Collaborative Filtering) that can learn arbitrary function from data by replacing the inner product [37]. Different from MFNN, a deep neural architecture is used to achieve the score for an interaction pair in NCF. Under NCF framework, they propose two instantiations: GMF (Generalized Matrix Factorization) that applies a linear kernel to learn the interaction function, in which the element-wise product of latent vector is projected to the output layer with a linear activation function; Another instantiation is MLP (Multi-Layer Perceptron) that employs a non-learner kernel to model the latent feature interactions, in which the interaction feature is fed into a multi-layer perceptron to learn the latent features of interaction pairs, and then is projected to the output layer. Finally, the last hidden layer of GMF and MLP is concatenated to build a fused model NeuMF (Neural Matrix Factorization). It is observed that MFNN is essentially also instantiation under NCF framework with different kernel model.

**Fig. 4:**
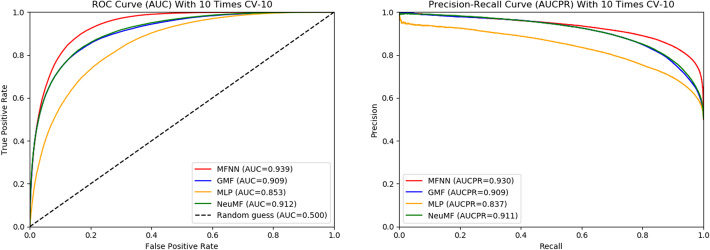
The ROC and Precision-Recall curves obtained for each model with 10 times CV-10

**Fig. 5:**
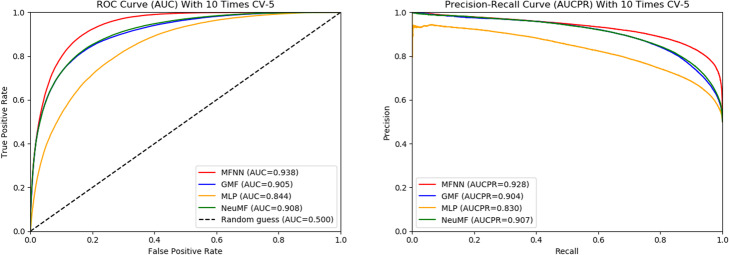
The ROC and Precision-Recall curves obtained for each model with 10 times CV-5

In this section, we compare the MFNN method with GMF, MLP and NeuMF models on the same dataset CRIM. The comparing results are shown in Fig. 4-5. Figure 4 shows that the ROC and Precision-Recall curves obtained for each model with 10 times CV-10. MFNN achieves the highest AUC and AUCPR values under 10 times CV-10, which is not obvious, only 0.02 higher than that of NeuMF. This may be because MFNN is essentially also instantiation under NCF framework. MLP has the lowest AUC and AUCPR values, like MFNN, its performance degrades as the network deepens. In addition, the fused model NeuMF achieves the higher AUC and AUCPR than CMF and MLP on dataset CRIM. The results of 10 times CV-5 are similar to CV-10 for each model, as shown in Fig. 5.

To further assess the performance of MFNN and the other models on the CRIM, we introduce the evaluation metrics including sensitivity (*S*_*n*_), specificity (*S*_*p*_), precision (*P*_*r*_), accuracy (*Acc*) and Matthew’s correlation coefficient (*MCC*). Analogously, two cross-validation are adopted to test the model performance, and the final values of these evaluation metrics are the average values with 10 times cross-validation results. The results are shown in Table 1.

**Table 1:** Assess results for each model with different validation methods

Validation methods	Methods	***S***_***n***_	***S***_***p***_	***P***_***r***_	***Acc***	***MCC***
10 times CV-10	MFNN	0.7905	0.9050	0.8928	0.8477	0.7003
	GMF	0.7262	0.9054	0.8849	0.8158	0.6422
	MLP	0.7149	0.8151	0.7951	0.7650	0.5333
	NeuMF	0.7327	0.9019	0.8821	0.8173	0.6441
10 times CV-5	MFNN	0.7905	0.9027	0.8906	0.8466	0.6978
	GMF	0.7209	0.9031	0.8816	0.8120	0.6347
	MLP	0.6968	0.8142	0.7899	0.7555	0.5150
	NeuMF	0.7262	0.9002	0.8795	0.8132	0.6364

In Table 1, *S*_*n*_ and *MCC* values are relatively low compared to the other metrics, however, MFNN is much higher than the other models in terms of *S*_*n*_ and *MCC*. *S*_*n*_ of MFNN is 0.05 higher than that of NeuMF under 10 times CV-10, meanwhile, MFNN also achieves 0.06 higher than NeuMF in term of *MCC*. Moreover, MFNN obtains the higher value in terms of *S*_*p*_, *P*_*r*_, and *Acc*. In conclusion, these evaluation metrics indicate that MFNN performs better than other models on circRNA-RBP dataset CRIM.

### Performance results

**Fig. 6:**
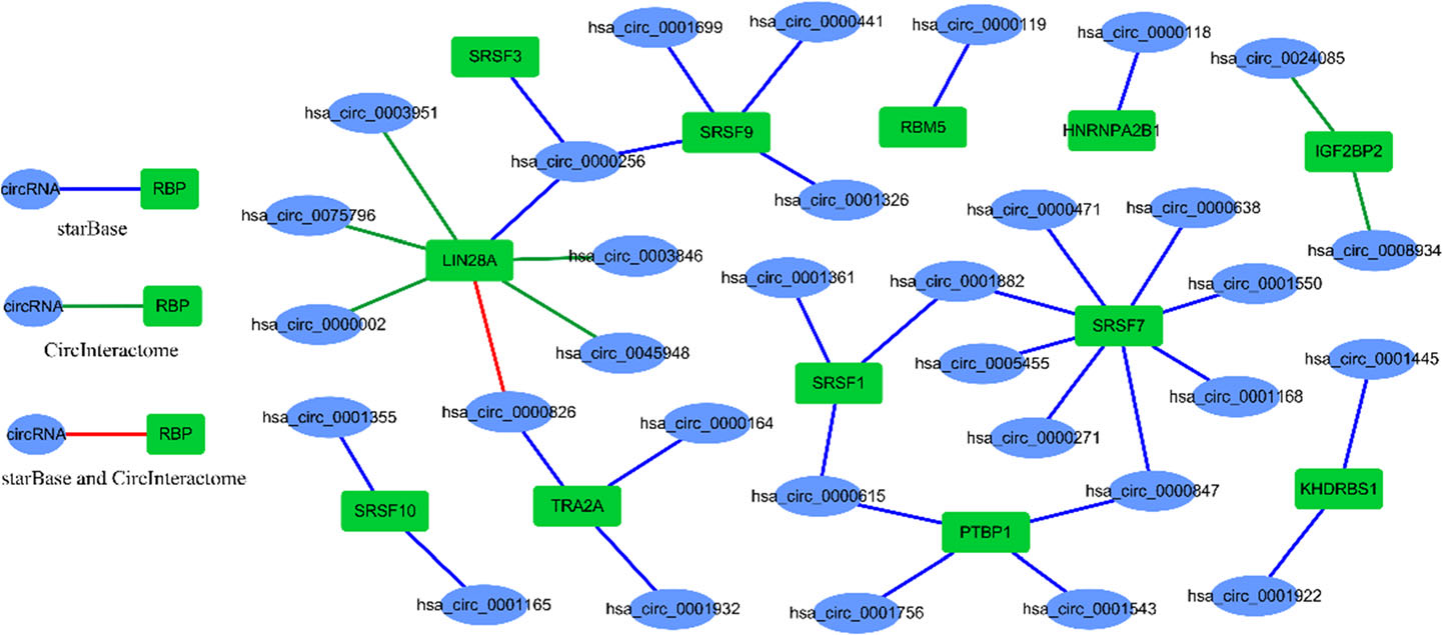
The predicted interaction networks with matching the other databases

In this section, kernel model MFNN is used to score the unlabeled samples with P-U learning. During the experiments, to ensure that any unlabeled sample is scored over 5 times by MFNN, the times of random sampling round is set to 10 according to Formula 11. Finally, the score of any unlabeled is calculated by averaging the results of MFNNs scores, all unlabeled samples are scored by this way. In this study, an interaction pair with score greater than 0.7 has high credibility including 662 interaction pairs. Then, to demonstrate the effectiveness of kernel MFNN, we apply the database starBase and CircInteractome to compare the 662 interaction pairs, the search results are shown in Table 2 and Fig. 6. The starBase and CircInteractome are marked S and C, respectively.

**Table 2:** The predicted interactions pairs recorded by other databases with score more than 0.7

pair	rank	score	database	pair	rank	score	database
SRSF9 hsa_circ_0001851	4	0.856	S	SRSF9 hsa_circ_0000256	362	0.735	S
SRSF7 hsa_circ_0000471	19	0.829	S	RBM5 hsa_circ_0000119	364	0.734	S
SRSF7 hsa_circ_0001168	45	0.809	S	TRA2A hsa_circ_0000826	404	0.729	S
SRSF9 hsa_circ_0000441	59	0.803	S	SRSF7 hsa_circ_0000638	408	0.728	S
TRA2A hsa_circ_0000164	63	0.802	S	LIN28A hsa_circ_0045948	411	0.727	C
SRSF7 hsa_circ_0000271	67	0.798	S	PTBP1 hsa_circ_0000847	462	0.721	S
SRSF10 hsa_circ_0001355	97	0.786	S	SRSF7 hsa_circ_0001550	472	0.720	S
SRSF1 hsa_circ_0000615	102	0.785	S	KHDRBS1 hsa_circ_0001922	480	0.719	S
SRSF9 hsa_circ_0001699	150	0.771	S	PTBP1 hsa_circ_0000615	492	0.719	S
PTBP1 hsa_circ_0001756	152	0.771	S	SRSF3 hsa_circ_0000256	511	0.717	S
SRSF7 hsa_circ_0000847	192	0.762	S	IGF2BP2 hsa_circ_0008934	519	0.716	C
SRSF10 hsa_circ_0001165	207	0.759	S	LIN28A hsa_circ_0003846	543	0.713	C
KHDRBS1 hsa_circ_0001445	259	0.750	S	LIN28A hsa_circ_0003951	559	0.711	C
IGF2BP2 hsa_circ_0024085	266	0.749	C	SRSF1 hsa_circ_0001882	566	0.710	S
LIN28A hsa_circ_0075796	291	0.744	C	HNRNPA2B1 hsa_circ_0000118	577	0.709	S
SRSF1 hsa_circ_0001361	294	0.743	S	TRA2A hsa_circ_0001932	592	0.708	S
SRSF7 hsa_circ_0001882	302	0.742	S	LIN28A hsa_circ_0000256	637	0.702	S
LIN28A hsa_circ_0000826	328	0.738	S, C	PTBP1 hsa_circ_0001543	654	0.701	S
LIN28A hsa_circ_0000002	337	0.737	C	SRSF9 hsa_circ_0001326	655	0.701	S
SRSF7 hsa_circ_0005455	359	0.735	S

Table 2 shows the results matching to the other databases with the interaction pairs of predicted by kernel MFNN. The interaction pair is listed in the first column of the table, the score of pair is given in the third column of the table. Then, the places of interaction pair within predicted results is shown in the second column. In addition, the fourth column is the matched database name. Due to the different RPBs are recorded in various database, intersection is less, only a few RBPs have been matched, especially in CircInteractome. Finally, 39 interaction pairs could be found in the other database including 12 RBPs and 33 circRNAs.

Figure 6 shows the newly predicted interactions which are extracted from starBase and CircInteractome. Blue and green lines indicate the interactions extracted from starBase and CircInteractome, respectively. Red lines represent the interactions recorded in the two databases, simultaneously. Moreover, Fig. 6 also exhibits that more RBPs are matched from starBase, this is probably because the RBP included in starBase has more overlap with our dataset. The network shows that SRSF7, SRSF1, SRSF9, PTBP1 and TRA2A sponge more circRNAs in predicted results. For example, SRSF7 interacts with 8 circRNAs in newly predicted interactions which is matched with the known interactions in starBase. LIN28A sponges 7 circRNAs as well, they are matched with the known interactions in CircInteractome. The hsa_circ_0000256 interacts with multiple RBPs. Moreover, the pair between LIN28A and hsa_circ_0000826 is matched with the known interaction in starBase and CircInteractome, simultaneously.

## Discussion

Increased evidence has shown that the interactions between circRNAs and RBPs are significant for many biological processes and human diseases, which are also deemed to an essential element underlying the functions of circRNA. Biological experimental remains some challenges such as cost-heavy, labor-intensive and time-consuming, designing an accurately computational method for predicting the circRNA-RBP interaction pairs could provide valuable supports for revealing the molecular mechanism within various biological processes.

In this study, we construct a matrix factorization framework based on neural networks to predict the circRNA-RBP interactions. The circRNA-RBP interactions are collected from CircRic database, Then, these data are transformed to interaction matrix as the input of our model. Due to lack of negative samples, the P-U learning strategy is employed to score the unlabeled samples. During the experiments, the best model is selected through analyzing the architecture and parameters of the MFNN model. Compared to the different deep kernel models, MFNN has an advantage in the prediction accuracy. Finally, the predicted interaction pairs are matched to the known interactions in the other databases. Results of the experiments show that MFNN is an effective model for analyzing the circRNA-RBP interactions. The better performance of MFNN is mainly attributed to the following aspects. Firstly, there is no need for additional circRNA and RBP biochemical characteristics in the prediction process, which not only simplifies the complexity of the model but also avoids the prediction bias caused by feature selection. Secondly, the hypothesis that similar circRNAs have similar interactions was discarded. Instead, latent interaction factors are mined by neural network acting on circRNA-RBP interactions.

Despite the effectiveness of the MFNN, it should be noted that MFNN still has some limitations. It is powerless for new circRNA or RBP which is the common shortcoming of matrix factorization method, and it is also a problem that we need to solve in the future. In addition, the prediction effect of our designed model is poor for circRNA or RBP with few known relationships, which is also a common fault of the recommendation system. We will make up for this by collecting more reliable interaction pairs in the future.

## Conclusion

For a poorly studied circRNA-RBP interactions, we constructed a prediction framework only based on interaction matrix employing matrix factorization and neural network. We demonstrate that MFNN achieves higher prediction accuracy, and it is an effective method. It can be further extended to predict other biological interaction links, such as circRNA and diseases, circRNA and miRNA, etc. We hope that our prediction model can contribute to further understanding of the functions of circRNA.

## Methods

**Fig. 7:**
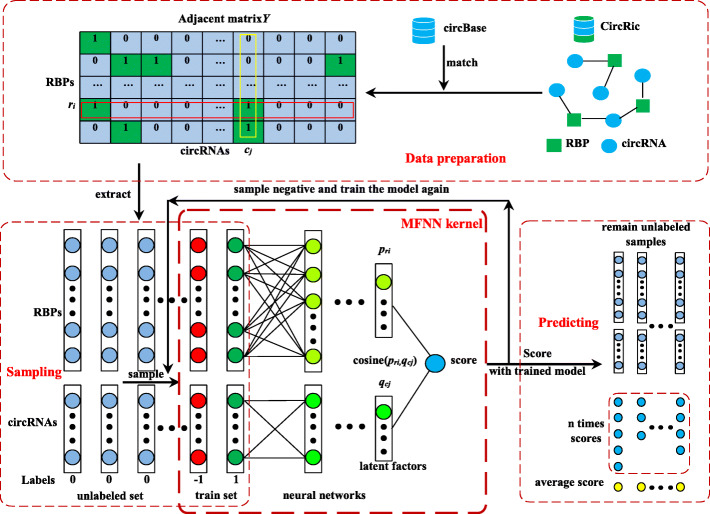
Schematic diagram of matrix factorization with neural network. 1) The circRNA-RBP interaction data is downloaded from the CircRic database, and the interaction matrix *Y* could be obtained by matching with the circRNA IDs in circBase database. 2) According to the P-U learning mechanism, negative samples with the same number of positive samples are randomly selected from the unlabeled relationships to obtain the training data set. 3) Based on the matrix classical factorization method, the neural network algorithm is used to obtain the latent factors of circRNAs and RBPs, and the scores of circRNA-RBP interactions are obtained by calculating the cosine of the latent factors. 4) The unlabeled relationships are scored using the trained model

In this study, we construct a matrix factorization-based prediction framework, namely, matrix factorization method with neural network architecture, to predict unknown circRNA-RBP interaction pairs by employing P-U learning. Here, neural network is employed to extract the latent factors of circRNA and RBP, and the P-U learning strategy is applied to predict unknown interaction pairs. For this purpose, the known circRNA-RBP interaction data are collected from CircRic [15], which form the dataset for training and testing the model, respectively. The Schematic diagram of model is shown as Fig. 7.

### Dataset

In recent years, although the studies of circRNA-RBP interaction are various, unfortunately, there are still no unified public datasets on circRNA-RBP interactions so far, especially in cancers. In this study, we construct a circRNA-RBP interactions matrix by using the public databases. Ruan et al. analyzed the association between circRNAs and proteins in 935 cancer cell lines across 22 cancer lineages from Cancer Cell line Encyclopedia (CCLE), and provides a data portal (CircRic, https://hanlab.uth.edu/cRic/) [15]. We download circRNA and RBP binding data from CircRic, to build the circRNA-RBP interaction matrix. To obtain a credible interaction matrix, the interaction pairs are preserved whose circRNA is saved by circRNA database circBase [41]. Finally, the interaction matrix contains 8473 interaction pairs with 94 circRNAs and 673 RBPs, namely, CRIM (circRNA-RBP Interaction Matrix) and serves as the input of our model.

### Notation

Consider a set of known circRNAs $C=\left\{{c}_1,{c}_2,\dots, {c}_{N_c}\right\}$ and known RBPs $R=\left\{{r}_1,{r}_2,\dots, {r}_{N_r}\right\}$, where *N*_*c*_ is the number of circRNAs and *N*_*r*_ is the number of RBPs, respectively. Let *Y* be an *N*_*r*_ × *N*_*c*_ adjacency matrix, which is an interaction matrix between circRNAs and RBPs. If a circRNA *c*_*j*_ interacts with a RBP *r*_*i*_ , *y*_*i*, *j*_ = 1 , otherwise *y*_*i*, *j*_ = 0. *F* is objective matrix, which is an *N*_*r*_ × *N*_*c*_ score matrix. The score *f*_*i*, *j*_ of *F* indicates the probability of interaction between RBP *r*_*i*_ and circRNA *c*_*j*_. In addition, in the P-U learning algorithm, the positive example set is noted as *P*, in which the score of interaction pairs *y*_*i*, *j*_ in adjacency matrix *Y* is 1, *U* indicates the unlabeled examples set and the score of interaction pairs *y*_*i*, *j*_ is 0.

### Model formulation

In this section, we construct a prediction model employing P-U learning with a matrix factorization framework based on neural networks (MFNN) for predicting unknown circRNA-RBP interaction pairs by using the interaction matrix *Y*. As shown in Fig. 7.

Matrix factorization methods are commonly used to solve the problem of estimating the scoring of each unknown entry in an interaction matrix *Y*, namely, the objective matrix *F*. Koren et al. [42] estimates the score *f*_*i*, *j*_ of *F* by calculating the dot product of interaction pairs (e.g. *p*_*ri*_, *q*_*cj*_ ) in Latent Factor Model (LFM). In this study, *p*_*ri*_ and *q*_*cj*_ is the latent representations of RBP *r*_*i*_ and circRNA *c*_*j*_, respectively. It can be described as follows:

6 $$ {f}_{i,j}={q_{cj}}^T{p}_{ri} $$

where *f*_*i*, *j*_ is the score of RBP *r*_*i*_ and circRNA *c*_*j*_ interaction, obviously, latent representation of circRNA and RBP is the key idea of this approach.

Hand-crafted features of interaction pairs may change the intrinsic feature distribution of the data and need rich professional theory knowledge. With the development of machine learning method, neural networks algorithms are often used to learn the latent features automatically. Xue et al. use a neural network to obtain the latent representations for a given interaction pair [38]. Inspired by this, neural network is employed to represent the circRNAs and RBPs in a latent low-dimensional space in this study. The latent representation of RBP *r*_*i*_ and circRNA *c*_*j*_ are given as follows:

7 $$ {p}_{ri}={f}^{\mathrm{l} ayer\_n}\left( map\left(\dots {f}^{layer\_1} map\left({y}_{i\ast },{w}_{r\_1}\right)\dots, {w}_{r\_n}\right)\right) $$

8 $$ {q}_{cj}={f}^{layer\_n}\left( map\left(\dots {f}^{layer\_1} map\left({y}_{\ast j},{w}_{c\_1}\right)\dots, {w}_{c\_n}\right)\right) $$

here, *y*_*i*∗_ is the *i-th* row of matrix *Y*, denotes the *i-th* the RBP scoring across all circRNAs. *w*_*r* _ *i*_ is the weighting parameters in the neural network *map*. *f*(*x*) is a non-linear activation function such as the Rectified Linear Unit (ReLU). *q*_*cj*_ is obtained through the similar formula. Finally, the score of an interaction pair is calculated using cosine distance between *p*_*ri*_ and *q*_*cj*_:

9 $$ {f}_{i,j}= cosine\left({p}_{ri},{q}_{cj}\right)=\frac{{q_{cj}}^T{p}_{ri}}{\left\Vert {p}_{ri}\right\Vert\ \left\Vert {q}_{cj}\right\Vert } $$

where ‖*p*_*ri*_‖ and ‖*q*_*cj*_‖ is the norm of *p*_*ri*_ and *q*_*cj*_, respectively.

In the model training phase, the binary cross-entropy loss is adopted as loss function:

10 $$ L=-\sum \limits_{\left(i,j\right)\in {Y}^{train}}{y}_{ij}\log {f}_{ij}+\left(1-{y}_{ij}\right)\log \left(1-{f}_{ij}\right) $$

where *y*_*ij*_ is the real label. Need not point out that *f*_*i*, *j*_ can be negative, in this study, the score is converted to a very small number such as 1.0*e*^−6^ if it is negative. The detail training and evaluating algorithm is described in Table 3.

**Table 3:** Procedure of the General MFNN algorithm

**Algorithm 1:** The General MFNN Algorithm	
**Input:***Y*: the known interaction matrix **Set:** Epoch: *e*, Batch size: *b*, Learning rate: *l*	
**Output:***W*: model parameters	
1: Randomly sample the train set *Y*^*train*^ and validation set *Y*^*vali*^ from *Y*.	
2: Initialize the model parameters *w*_*c* _ *n*_ and *w*_*r* _ *n*_ with a Gaussian distribution	
3: **while not** model is converged and epoch > e **do**	
sample a mini batch from *Y*^*train*^ in size *b*	
set *p*_*ri*_ and *q*_*cj*_ using Eq. 2 and 3 with mini batch	
set *f*_*i*, *j*_ using Equation 4 with *p*_*ri*_ and *q*_*cj*_	
set *L* using Equation 5 with *f*_*i*, *j*_ and *y*_*ij*_	
use Adam optimizer to optimize model parameters	
**end while**	
4: using the *Y*^*vali*^ evaluate the model	

Due to the lack of negative samples in CRIM, only positive and unlabeled samples, the standard supervised learning method is no longer applicable. Generally, the problem of learning a binary classifier from a training set of positive and unlabeled samples refers as P-U learning [39]. It turns the problem into discriminating *P* from random subsamples of *U* by creating a series of classifiers, then, each of these classifiers assigns a prediction score to any unlabeled sample, the final prediction score for any unlabeled sample is the average score of the individual classifiers, Inspired by this, the P-U learning is applied to solve the problem of CRIM data imbalance.

In this study, the classifiers are a set of MFNN trained with the dataset CRIM. The detail procedure is described in Table 4. In each round, *U*_*t*_ which has the same size as *P* is random subsamples of *U*, the model MFNN is trained with the training set by *U*_*t*_ and *P* composition, then it is used to score the unlabeled samples in *UU*_*t*_. In this way, no sample of *U* is used simultaneously to train and test the model MFNN. Finally, the score of any sample *u* in *U* is voted by averaging the predictions of the MFNNs which is trained on subsamples without sample *u*. To obtain the final score of unlabeled sample *u*, the counter *t*(*u*) is introduced to count the times of unlabeled sample *u* predicted by the classifier MFNN. In particular, according to experimental experiences of Mordelet et [39]., to ensure that any unlabeled sample *u* is scored over *n* times by MFNN, the times of sampling round *T* is chosen as Formula 11.

11 $$ T\left(1-\frac{K}{\left|U\right|}\right)\ge n $$

where *K* is the sampling-size. |*U*| is the number of unlabeled samples. In this study, according to the size of CRIM and unlabeled set *U*, *T* is set to 10.

**Table 4:** Procedure of the MFNN with P-U learning algorithm

**Algorithm 2:** The MFNN with P-U learning Algorithm	
**Input:***Y*: the known interaction matrix, *T*: the times of sampling round **Set:** Obtain set *P* and *U* from *Y*, *K*: the size of *P* in each sampling round	
**Output:***F*_*u*_: unlabeled sample score	
Step 1: Initialize ∀*u* ∈ *U*, *t*(*u*) ← 0, *MFNN*(*u*) ← 0	
Step 2: **For** *t* from 1 to *T* **do**	
Randomly sample the set *U*_*t*_ of size *K* in *U*.	
Train a model *MFNN*_*t*_ to discriminate *P* against *U*_*t*_	
For ∀*u* ∈ *U*\*U*_*t*_, update:	
*MFNN*(*u*) ← *MFNN*(*u*) + *MFNN*_*t*_(*u*)	
t (*u*) ← *t*(*u*) + 1	
**end For**	
Step 3: Return *F*_*u*_ = *MFNN*(*u*)/*t*(*u*) for *u* ∈ *U*	

## Data Availability

The datasets used and/or analyzed during the current study are available from the corresponding author on reasonable request.

## Abbreviations

circRNA: Circular RNA
RBP: RNA Binding Protein
P-U learning: Positive Unlabeled learning
MFNN: Matrix Factorization with Neural Networks
CRIM: CircRNA-RBP Interaction Matrix
GMF: Generalized Matrix Factorization
MLP: Multi-Layer Perceptron
NeuMF: Neural Matrix Factorization
